# Short-term effects of non-grazing on plants, soil biota and aboveground-belowground links in Atlantic mountain grasslands

**DOI:** 10.1038/s41598-017-15345-1

**Published:** 2017-11-08

**Authors:** Lur Epelde, Anders Lanzén, Iker Mijangos, Estibaliz Sarrionandia, Mikel Anza, Carlos Garbisu

**Affiliations:** 1 NEIKER-Tecnalia, Basque Institute of Agricultural Research and Development, Department of Conservation of Natural Resources, c/Berreaga 1, E-48160 Derio, Spain; 2 Department of Plant Biology and Ecology, University of the Basque Country (UPV/EHU), E-01006 Vitoria, Gasteiz Spain

## Abstract

Mountain grasslands in the Iberian Peninsula are the result of extensive grazing. However, a progressive abandonment of grazing activity is currently observed in the study region. The objective of this work was to evaluate the short-term (2 years) effects of non-grazing on the diversity and composition of plants, soil microorganisms (prokaryotes, fungi, arbuscular mycorrhiza), mesofauna, macrofauna and aboveground-belowground links, through the study of 16 grazed *vs*. non-grazed areas in Atlantic grasslands located in the Basque Country (Spain). Sites were divided between 4 habitat types with different elevation, pasture productivity, vegetation type and parent material. Herbivores appeared to influence plant community composition, contributing to increase aboveground diversity, while having unequal effects on belowground communities depending on the organisms analysed. This may be explained by the different habitat and trophic level of each soil organism, which may be more or less affected by the predominating negative effects of grazing, such as soil compaction, and only partially compensated by other positive effects. Finally, habitat type appeared to be the strongest influence on both above- and belowground communities, also influencing the effect of the absence of grazing.

## Introduction

In mountain grasslands of the Iberian Peninsula, grazing has been the most important economic activity since the Neolithic^1^. These semi-natural grasslands deliver many ecosystem services, such as primary productivity and nutrient cycling, food production, preservation of genetic diversity, water storage, pest regulation, pollination, recreation, etc. However, noteworthy changes in management practices have taken place in these extensive livestock systems, including shorter grazing time in higher mountain pastures, lower stocking rate and less shepherd control^2^. These trends have also been detected in many parts of Europe; if they continue, a progressive medium- or long-term grazing abandonment is expected^3^. In particular, in the Basque Country, management practices are leading to the abandonment of grazing activity in many mountain areas.

Belowground responses to livestock grazing are less widely investigated than aboveground responses^2,4,5^. In this respect, soil microbial communities are known to play a crucial role in soil processes and the delivery of vital ecosystem services^6,7^; besides, they can influence plant community composition and productivity^8^. Among soil fungi, arbuscular mycorrhiza are thought to help plants tolerate grazing^9^. On the other hand, the activity and composition of soil microbial communities can also be influenced by (i) predator-prey interactions with small soil invertebrates and (ii) habitat formation due to macrofaunal activity^10^. These interactions are strongly influenced by, and may react in unison to, the presence of large herbivores. Herbivores exert this influence by (i) regulating the quantity and quality of organic matter and resource inputs, (ii) altering the functional composition of vegetation and (iii) physical disturbance^11^. These effects are likely to change depending on the edaphoclimatic conditions present in the grazed area, which, in turn, affect the structure and function of the biological communities present in each site. Unfortunately, there is little research and data integration regarding above-belowground responses and links, and few studies have simultaneously investigated the effect of grazing/non-grazing on plant species composition, soil macrofauna, mesofauna, microorganisms, and soil abiotic properties^12^. In this respect, one of the most significant challenges currently faced by ecologists is probably the enormous complexity and heterogeneity of the soil foodweb and its micro-habitats^13^, although technological advancements in DNA sequencing have opened up for large-scale studies of microbial community structure.

The main objective of this study was to assess the short-term (2 years) effects of non-grazing on plant communities, soil biological communities and the aboveground-belowground links between these two communities in Atlantic mountain grasslands. We hypothesized that cessation of grazing would either decrease or increase belowground activity and diversity in different ways for different soil organisms. Increased diversity may indicate negative grazing effects from compaction, while a decrease may indicate positive effects from e.g. increased availability of labile carbon due to fecal matter from grazers and increased production of plant exudates due to compensatory growth^11^. Which of these effects will dominate would depend on the habitat and trophic position of the community studied. Climatic and edaphic factors will also influence the effects^12^, with less fertile soils being less likely to show positive effects from grazing^11^.

Specific questions investigated in this study were: (Q1) Does cessation of grazing affect plant communities and various soil biological communities differently? (Q2) Are the links between these communities different in the presence *vs*. the absence of grazing? (Q3) Do the abovementioned effects vary depending on bedrock and habitat type, with a special focus on the fertility of the site? (Q4) Do traditional methods, such as community-level physiological profiles (CLPPs) and arbuscular mycorrhizal (AM) spore morphotyping, provide similar responses as those offered by molecular techniques, such as DNA sequencing, regarding the effects studied? To this end, we compared grazed to non-grazed sites in an Atlantic grassland area, performing a comprehensive analysis of soil biological communities through the determination of (i) basal and substrate-induced respiration; (ii) types of macrofauna; (iii) the “Qualitá Biologica del Suelo” – Soil Biological Quality (QBS) from mesofauna communities; (iv) community-level physiological profiles (CLPPs) of cultivable bacteria through Biolog Ecoplates^TM^; (v) diversity of morphotypes of AM spores; and (vi) prokaryotic and fungal diversity through 16 S rRNA and ITS amplicon sequencing, respectively. In total, grassland sites belonging to four distinct categories were studied (i.e., mountain and valley grasslands, each on siliceous as well as on calcareous bedrock) in order to cover the variability existing in the studied area.

## Results

Relatively infertile mountainous sites with high soil acidity and a pH between 3.9 and 5.5 dominated the sites studied, while soil pH ranged between 5.6 and 6.9 in more fertile valley sites. None of the chemical parameters related to soil nutrient levels (*i.e*., soil organic matter-SOM, total nitrogen, Olsen phosphorus and extractable potassium) nor pH were significantly affected by grazing (Table 1). However, soil compaction decreased significantly (39% in average) in the absence of grazing and, concomitantly, infiltration time significantly decreased (48% in average). In turn, soil humidity significantly increased (12% in average) in the absence of grazing (Table 1).

**Table 1:** Physicochemical parameters (average ± standard deviation; n = 4).

		6230a-NG	6230a-G	6170-NG	6170-G	6230c-NG	6230c-G	valley-NG	valley-G	*t-value*	*p-value*
SOM	(%)	17.9 ± 6.3	20.2 ± 5.0	9.0 ± 0.5	8.6 ± 1.4	10.6 ± 1.6	13.4 ± 0.4	5.1 ± 1.0	5.1 ± 0.7	*−1.145*	*0.263*
Total N	(%)	0.91 ± 0.21	0.91 ± 0.08	0.59 ± 0.03	0.52 ± 0.08	0.51 ± 0.06	0.55 ± 0.03	0.41 ± 0.06	0.39 ± 0.04	*0.398*	*0.694*
Olsen P	(mg kg^−1^)	8.8 ± 4.6	12.0 ± 7.9	4.8 ± 0.8	3.7 ± 1.6	13.8 ± 7.1	19.3 ± 9.6	26.2 ± 32.2	25.6 ± 33.4	*−0.345*	*0.733*
Extractable K^+^	(mg kg^−1^)	126 ± 29	146 ± 27	172 ± 38	107 ± 59	124 ± 34	87 ± 20	105 ± 47	203 ± 135	*−0.166*	*0.870*
pH		4.1 ± 0.1	4.1 ± 0.2	5.1 ± 0.2	4.8 ± 0.1	4.4 ± 0.1	4.2 ± 0.2	5.9 ± 0.3	6.3 ± 0.5	*0.161*	*0.874*
Compaction	(Mpa)	1422 ± 191	2038 ± 278	2051 ± 334	2854 ± 602	1373 ± 459	2337 ± 221	609 ± 213	1649 ± 333	*−6.887*	*0.000*
Infiltration time	(min)	7.8 ± 5.3	7.0 ± 1.8	2.4 ± 1.7	10.5 ± 13.3	13.9 ± 13.4	17.9 ± 8.4	7.5 ± 5.7	25.3 ± 8.5	*−2.315*	*0.029*
Soil humidity	(%)	51 ± 6	46 ± 5	23 ± 1	22 ± 1	28 ± 1	27 ± 4	26 ± 7	20 ± 2	*2.470*	*0.020*

In the right (italics), summary of the linear mixed-effect models.

G = grazed; NG = non-grazed.

Regarding biological parameters (Table 2), root depth and root abundance were not significantly affected by grazing. However, the number of macrofauna types and CO_2_ emission values increased significantly in the absence of grazing (39% and 36% in average, respectively), while the mesofauna QBS index decreased significantly (15% in average).

**Table 2:** Biological parameters (average ± standard deviation; n = 4).

		6230a-NG	6230a-G	6170-NG	6170-G	6230c-NG	6230c-G	valley-NG	valley-G	*t-value*	*p-value*
Root depth	(cm)	23 ± 5	23 ± 5	19 ± 3	18 ± 5	26 ± 3	24 ± 5	16 ± 3	14 ± 5	*1.025*	*0.315*
Root abundance	(index: 1–10)	9.5 ± 0.6	9.5 ± 0.6	7.8 ± 1.0	6.8 ± 1.0	8.1 ± 0.5	8.3 ± 0.5	7.1 ± 0.9	6.4 ± 1.3	*1.616*	*0.118*
Macrofauna types	(number)	2.8 ± 1.5	1.5 ± 1.3	3.0 ± 1.4	2.5 ± 0.6	3.0 ± 1.4	3.0 ± 1.2	6.5 ± 1.7	4.0 ± 0.8	*2.247*	*0.033*
Mesofauna	(index)	55 ± 17	66 ± 18	36 ± 10	45 ± 6	54 ± 4	63 ± 5	53 ± 8	58 ± 16	*−2.235*	*0.034*
CO_2_ emissions	(g CO_2_ m^−1^ h^−1^)	1.0 ± 0.2	0.7 ± 0.1	1.2 ± 0.3	1.2 ± 0.2	2.1 ± 0.4	1.3 ± 0.2	1.7 ± 0.6	1.2 ± 0.3	*3.434*	*0.002*
Basal respiration	(mg C-CO_2_ kg^−1^ h^−1^)	4.9 ± 1.2	4.7 ± 1.0	2.3 ± 0.4	1.9 ± 0.3	2.1 ± 0.4	2.2 ± 0.4	2.0 ± 0.7	1.3 ± 0.3	*1.211*	*0.237*
SIR	(mg C-CO_2_ kg^−1^ h^−1^)	28 ± 4	31 ± 3	24 ± 5	20 ± 4	13 ± 4	16 ± 3	16 ± 3	16 ± 2	*−0.495*	*0.624*

In the right (italics), summary of the linear mixed-effect models.

SIR = substrate-induced respiration; G = grazed; NG = non-grazed.

The diversity indexes for all community composition datasets are listed in Table 3. In all plots, a total of 73 different plant species, 22,219 prokaryotic OTUS, 8,037 fungal OTUs and 18 AM spore morphotypes were identified. Plant richness and Shannon’s diversity index decreased significantly in the absence of grazing, while prokaryotic richness instead increased significantly. Other diversity indexes, *i.e*. fungal and glomeromycota diversity (from ITS amplicon sequencing data), CLPPs of bacteria and AM spore morphotypes, did not change significantly. However, when performing this analysis considering only the more infertile mountainous sites, the richness of AM spore morphotypes increased significantly in the absence of grazing (t-value = 2.212, p-value = 0.038). Instead, when considering only the valley sites, Shannon’s diversity from CLPPs of bacteria decreased significantly (t-value = −2.777, p-value = 0.039).

**Table 3:** Biodiversity indexes in different community datasets (average ± standard deviation; n = 4).

		6230a-NG	6230a-G	6170-NG	6170-G	6230c-NG	6230c-G	valley-NG	valley-G	*t-value*	*p-value*
Plants	S	14 ± 3	16 ± 2	17 ± 4	20 ± 4	11 ± 3	13 ± 1	13 ± 3	17 ± 3	*−2.609*	*0.015*
	H’	2.06 ± 0.32	2.16 ± 0.21	2.13 ± 0.41	2.47 ± 0.16	1.56 ± 0.57	1.96 ± 0.17	1.92 ± 0.40	2.01 ± 0.29	*−2.066*	*0.049*
Prokaryotes	S	3241 ± 197	3067 ± 317	4842 ± 383	4417 ± 229	3273 ± 325	3371 ± 399	5464 ± 330	5194 ± 331	*2.958*	*0.007*
	H’	6.56 ± 0.06	6.45 ± 0.08	6.66 ± 0.11	6.59 ± 0.08	6.48 ± 0.05	6.53 ± 0.12	7.32 ± 0.06	7.32 ± 0.09	*1.630*	*0.115*
Fungi	S	614 ± 80	527 ± 85	1216 ± 114	986 ± 178	646 ± 119	666 ± 99	1248 ± 36	950 ± 256	*1.787*	*0.086*
	H’	3.50 ± 0.37	3.44 ± 0.41	4.56 ± 0.14	4.43 ± 0.26	3.82 ± 0.35	3.76 ± 0.32	4.78 ± 0.38	4.41 ± 0.52	*0.756*	*0.457*
Glomeromycota	S	17 ± 3	18 ± 5	43 ± 6	32 ± 14	26 ± 4	23 ± 2	64 ± 11	58 ± 6	*1.790*	*0.085*
	H’	1.90 ± 0.36	1.97 ± 0.29	2.82 ± 0.25	2.55 ± 0.45	2.48 ± 0.27	2.31 ± 0.14	3.56 ± 0.30	3.43 ± 0.22	*1.177*	*0.250*
CLPPs bacteria	S	16 ± 1	17 ± 2	21 ± 2	19 ± 3	18 ± 1	18 ± 2	22 ± 2	25 ± 2	*−0.505*	*0.618*
	H’	3.57 ± 0.29	3.67 ± 0.27	4.17 ± 0.11	3.84 ± 0.34	3.92 ± 0.06	3.89 ± 0.24	4.16 ± 0.17	4.42 ± 0.13	*−0.146*	*0.885*
AM spore morphotypes	S	14 ± 1	13 ± 2	14 ± 1	11 ± 2	13 ± 1	13 ± 2	15 ± 2	15 ± 3	*1.648*	*0.111*
	H’	2.59 ± 0.25	2.59 ± 0.13	2.43 ± 0.23	2.29 ± 0.29	2.64 ± 0.16	2.39 ± 0.28	2.13 ± 0.59	2.14 ± 0.39	*0.919*	*0.367*

In the right (italics), summary of the linear mixed-effect models.

G = grazed; NG = non-grazed.

Supplementary Figure S1 shows the distribution of the 20 most abundant (A) plant, (B) prokaryotic and (C) fungal taxa detected. Plant communities appeared to vary considerably both between mountain *vs*. valley plots and between non-grazed *vs*. grazed plots (Supplementary Figure S1A). Indeed, the abundance (percentage cover) of several plant species changed significantly in the absence of grazing (increasing for *Brachypodium pinnatum*, *Erica vagans*; and decreasing for *Agrostis curtisii*, *Trifolium repens*, *Danthonia decumbens*, *Carex caryophillea*) (Supplementary Table S1). Instead, prokaryotic communities varied less between habitats at the order level and none of the top 20 orders showed significant differences when comparing grazed *vs*. non-grazed plots (Supplementary Figure S1B). Fungal communities were less even at order level (Supplementary Figure S1C) and the most abundant taxa differed greatly depending on habitat. For example, Archaeorhizomycetales constituted 36 and 33% in 6230a and 6230c mountain habitats, respectively, but only 6 and 4% in 6170 mountain and valley plots, respectively. Further, Mortierelalles constituted 23, 21 and 27% in 6170, 6230c and valley plots, respectively, but only 4% in 6230a habitat. Finally, Agaricales constituted 11 and 8% in 6170 and valley plots, respectively, but only 1% in 6230a and 6230c habitats. From these top 20 orders, only Chaetothyriales and Eurotiales showed significantly higher relative abundance values in the absence of grazing.

**Figure 1:**
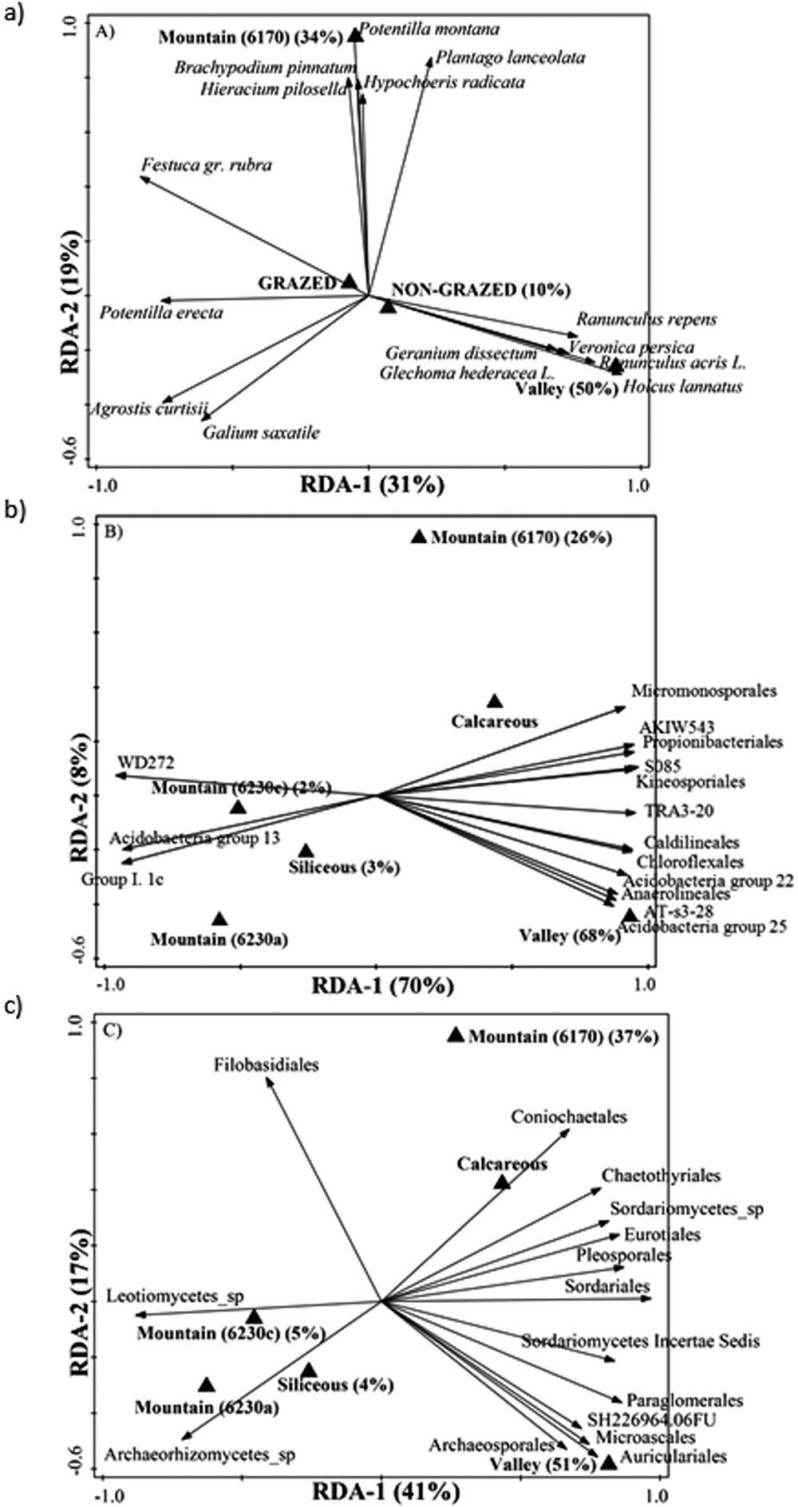
Biplot of the redundancy analysis performed with (**a**) plant community composition, (**b**) prokaryotic community composition at the order level, and (**c**) fungal community composition at the order level, as response variables, with grazing, bedrock and habitat as explanatory variables. Only the 15 response variables with best fit are shown. Variation explained by each axis is indicated between brackets. The explanatory variables that appear are those that significantly explained the variation in response data following forward selection; the contribution percentage of each variable is shown between brackets.

In order to study the effect of grazing at the community composition level, pCCAs were performed with datasets of plants, CLPPs of bacteria, AM spore morphotypes, and amplicon sequence data of 16S rRNA and ITS, including grazing as an explanatory variable and site as a covariable. Out of these analyses, only that using plant community composition as response variable showed statistically significant effects according to the Monte Carlo permutation test (Supplementary Fig. S2). Therefore, we proceeded to study the effect of other variables besides grazing (such as habitat and bedrock) on community composition. Valley habitats (compared to others) accounted for 50% of the explained variation in plant composition, while the Type 6170 mountain habitat accounted for 34% of the explained variation (pseudo-F = 7.3, p-value = 0.002; Fig. 1a). These two habitats presented a fairly different plant community composition. Instead, the effect of grazing, even if statistically significant, was limited to 10% of the explained variation. Both for prokaryotes (Fig. 1b; pseudo-F = 23.7, p-value = 0.002) and fungi (Fig. 1c; pseudo-F = 9.4, p-value = 0.002), most of the explained variation in community composition data was also attributable to habitat type. As with plant communities, the valley and Type 6170 habitats accounted for the largest shares of the explained variation. Grazing was not significant in explaining variation for neither prokaryotic nor fungal community composition. Further, RDAs indicated higher prokaryotic and, to a lesser extent, fungal richness in valley plots.

**Figure 2:**
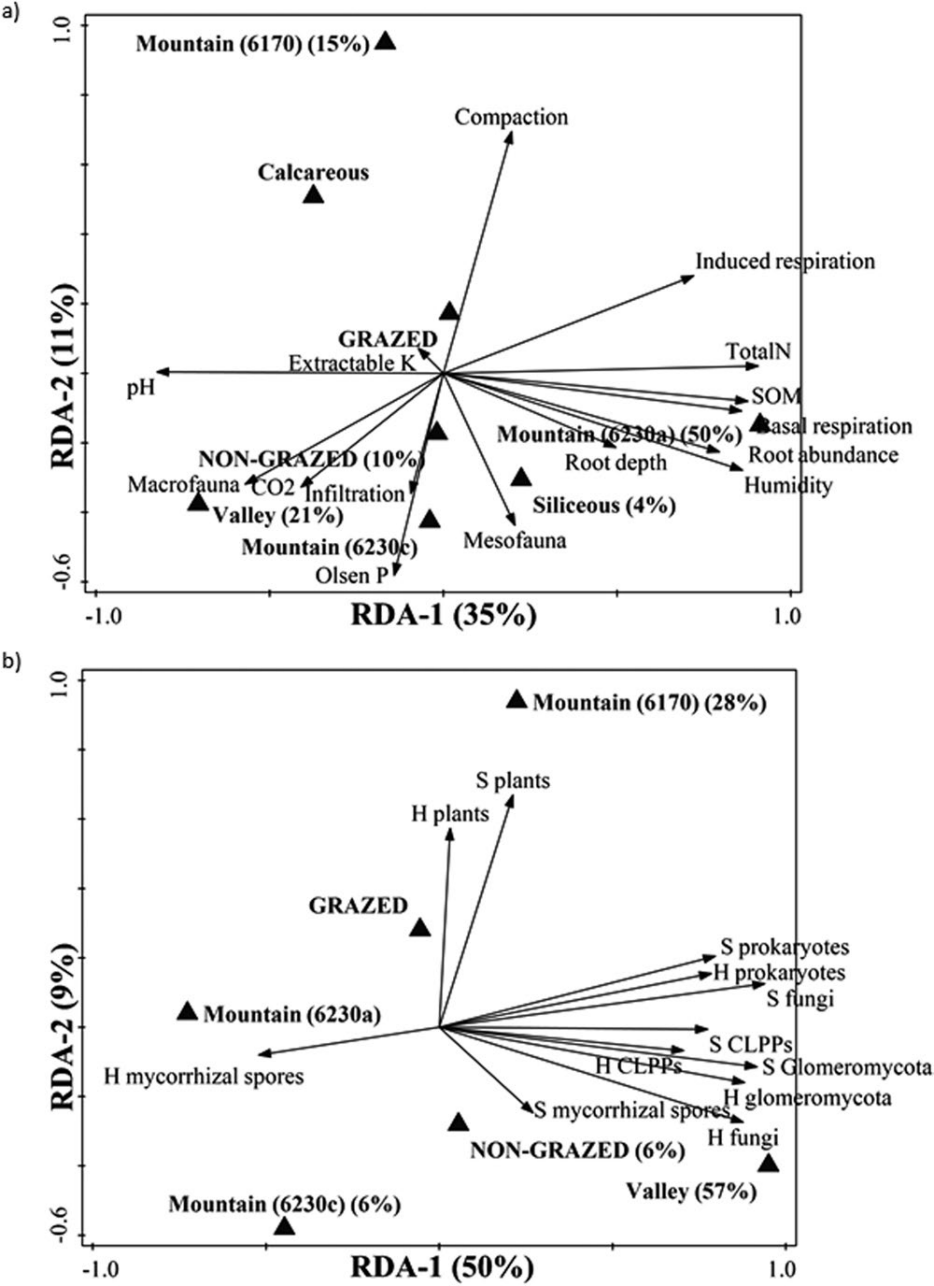
Biplot of the redundancy analysis performed with (**a**) physicochemical and biological parameters measured, and (**b**) all diversity indexes calculated, as response variables, with grazing, bedrock and habitat as explanatory variables. Variation explained by each axis is indicated between brackets. The explanatory variables that appear are those that significantly explained the variation in response data following forward selection; the contribution percentage of each variable is shown between brackets.

Supplementary Figure S3 show similar RDA plots performed with Glomeromycota community composition at the species level, CLPPs of bacteria and AM spore morphotype abundances. These three community datasets coherently agree with the results obtained with the broader and deeper amplicon sequence analyses, pointing mainly at valley habitat as the variable explaining most of the data variation and as the habitat that showed higher richness values. Also, grazing contributed significantly to 15% to the variation of CLPPs of bacteria obtained from Biolog EcoPlates^TM^.

Other soil physicochemical and biological parameters analyzed here are shown in Fig. 2a (pseudo-F = 10, p-value = 0.002). Most of the variation in these parameters was due to type of habitat, although also grazing regime and type of bedrock were significant variables. Soil respiration (basal and substrate-induced), total nitrogen, SOM, root depth and abundance, and soil humidity positively correlated with the 6230a mountain habitat, and pH was negatively correlated. When including all community diversity indexes as response variables (Fig. 2b; pseudo-F = 9.6, p-value = 0.002), the type of habitat (valley in particular) accounted for the greatest variability, but grazing was also significant. All belowground diversity indexes calculated here, except for H’ of AM spore morphotypes, were positively associated towards the positive region of RDA-1 and the valley habitat. Instead, plant diversity indexes were positively associated towards the positive region of RDA-2, the mountain 6170 habitat and the grazed sites.

The compositions of almost all community datasets correlated strongly with each other, as indicated by Mantel tests (Supplementary Table S2). Further, the strength of correlations did not change to a great extent depending on whether only grazed or non-grazed plots were considered. However, if considering diversity indexes instead of community composition, only belowground communities correlated significantly to each other (Table 4). The only exceptions to this was plant richness correlating with prokaryotic richness in grazed plots and plant richness with AM spore morphotype richness in non-grazed plots. In general, diversity indexes obtained from CLPPs correlated significantly with those obtained from amplicon data. With few exceptions, the diversity of AM spore morphotypes did not correlate significantly with any other parameter.

**Table 4:** Pearson’s correlations to compare richness (S) and Shannon’s (H’) diversity for every composition dataset.

				Plants	Prokaryotes	Fungi	Glomeromycota	CLPPs bacteria	AM spore morphotypes	
All plots	Plants	Pearson’s correlation	S		0.067	−0.020	−0.077	−0.229	0.100	H’
		p-value			0.715	0.912	0.675	0.207	0.586	
	Prokaryotes	Pearson’s correlation		0.260		**0.629**	**0.671**	**0.572**	−0.330	
		p-value		0.151		**0.000**	**0.000**	**0.001**	0.065	
	Fungi	Pearson’s correlation		0.285	**0.899**		**0.831**	**0.666**	**−0.419**	
		p-value		0.113	**0.000**		**0.000**	**0.000**	**0.017**	
	Glomeromycota	Pearson’s correlation		0.154	**0.769**	**0.888**		**0.708**	−0.340	
		p-value		0.400	**0.000**	**0.000**		**0.000**	0.057	
	CLPPs bacteria	Pearson’s correlation		0.107	**0.672**	**0.761**	**0.754**		−0.249	
		p-value		0.561	**0.000**	**0.000**	**0.000**		0.170	
	AM spore morphotypes	Pearson’s correlation		0.082	0.157	0.263	**0.399**	0.255		
		p-value		0.654	0.392	0.146	**0.024**	0.159		
Only grazed	Plants	Pearson’s correlation	S		0.174	−0.248	−0.157	−0.246	0.194	H’
		p-value			0.519	0.354	0.560	0.358	0.471	
	Prokaryotes	Pearson’s correlation		**0.506**		0.581	**0.582**	0.469	−0.302	
		p-value		**0.046**		0.018	**0.018**	0.067	0.255	
	Fungi	Pearson’s correlation		0.415	**0.835**		**0.902**	**0.822**	−0.397	
		p-value		0.110	**0.000**		**0.000**	**0.000**	0.128	
	Glomeromycota	Pearson’s correlation		0.279	**0.694**	**0.869**		**0.774**	−0.343	
		p-value		0.295	**0.003**	**0.000**		**0.000**	0.194	
	CLPPs bacteria	Pearson’s correlation		0.174	**0.654**	**0.806**	**0.863**		−0.129	
		p-value		0.519	**0.006**	**0.000**	**0.000**		0.633	
	AM spore morphotypes	Pearson’s correlation		−0.102	−0.074	0.156	0.412	0.380		
		p-value		0.708	0.784	0.563	0.113	0.147		
Only non-grazed	Plants	Pearson’s correlation	S		0.084	0.155	0.012	−0.262	0.132	H’
		p-value			0.757	0.566	0.965	0.327	0.626	
	Prokaryotes	Pearson’s correlation		0.310		**0.678**	**0.737**	**0.716**	−0.397	
		p-value		0.243		**0.004**	**0.001**	**0.002**	0.127	
	Fungi	Pearson’s correlation		0.290	**0.961**		0.767	0.465	−0.468	
		p-value		0.276	**0.000**		0.001	0.070	0.068	
	Glomeromycota	Pearson’s correlation		0.168	**0.822**	**0.902**		**0.657**	−0.373	
		p-value		0.535	**0.000**	**0.000**		**0.006**	0.155	
	CLPPs bacteria	Pearson’s correlation		−0.007	**0.840**	**0.775**	**0.693**		−0.388	
		p-value		0.980	**0.000**	**0.000**	**0.003**		0.138	
	AM spore morphotypes	Pearson’s correlation		**0.685**	0.331	0.414	0.381	0.060		
		p-value		**0.003**	0.211	0.111	0.145	0.826		

Bold: statistically significant results (p-value < 0.05).

## Discussion

### Effect of grazing on soil parameters, diversity indexes and composition (Q1)

Two years after establishing the non-grazed areas, no consequences were observed at the soil nutrient level. However, the reduction of soil compaction in the absence of grazing was very clear, explaining its higher water infiltration capacity. In fact, soil compaction was severe in some grazed areas (*i.e*., a pressure of over 3 MPa was necessary to penetrate the soil; data not shown). This suggests that the effect of grazing was largely negative, in accordance to Bardgett & Wardle^11^ for relatively infertile soils. Moreover, a higher rate of CO_2_ emissions was observed in non-grazed areas. This is also consistent with a previous study were grazing abandonment was also simulated by fencing; after five years, soil compaction decreased, values of enzyme activities and microbial biomass were reduced, and CO_2_ emission values increased^2^. Jensen *et al*.^14^ also detected a decrease in CO_2_ emissions due to compaction in a grazed soil and in a soil dedicated to corn production. In our case, this increase in non-grazed areas could be due to both increased circulation of water and gases through the soil and the accumulation of plant necromass. However, Klumpp *et al*.^15^ showed that grassland systems heavily disturbed through grazing foster soil carbon loss.

In our study, macrofauna richness increased in non-grazed areas, but the mesofauna QBS index decreased. Related results of grazing-induced effects on soil biota abundance and activity can be found in the literature: in a study by Bardgett *et al*.^16^, the absence of grazing decreased the abundance of microarthropods. Bardgett and Leemans^17^ also showed that two years without grazing reduced soil microbial biomass and activity, due to the cessation of liming on ungrazed grassland, but also due to the removal of readily utilizable substrate in the form of sheep excreta and urine and changes in the quantity and quality of root exudates.

The increase in percentage cover of *Erica vagans* in the absence of grazing may indicate an incipient succession towards heathland already after two years of exclosure. Apart from that, the increase of the unpalatable *Brachypodium pinnatum* in ungrazed grasslands has been known for a long time (*e.g*., Lousley^18^). Besides, this species belongs to a group of grasses that can dramatically reduce biodiversity as it colonizes the territory^19^. This might partly explain the lower plant richness and diversity in the absence of grazing, as well as the lower percentage cover of species like *Agrostis curtisii*, *Trifolium repens*, *Danthonia decumbens* and *Carex caryophillea*.

Despite the decrease in plant diversity, prokaryotic richness increased in the absence of grazing as did the abundance of some fungal taxa (*e.g*., Chaetothyriales and Eurotiales). By contrast, Aldezabal *et al*.^2^ observed no significant differences in bacterial richness between excluded and grazed plots. However, these authors used DGGE community profiling, which is generally less sensitive to changes in community diversity than the method used here. The increase in microbial diversity observed here may be related to the reduction of soil compaction and the higher values of water infiltration capacity found in the absence of grazing. Another reason could be the cessation of vegetation removal and, hence, the increase of litter accumulation in non-grazed areas. Changes in organic matter dynamics and nutrient supply are likely to have a profound influence on microbial community structure, as well as on plant physiological responses to grazing such as changes in root exudates or plant litter quality^20,21^.

As opposed to alpha diversity, we could not detect any significant effect of grazing on soil prokaryotic and fungal community composition. Soil is very heterogeneous, and each microorganism is circumscribed by its own immediate environment^22^. It might be that indirect changes that occur at a macroscale, such as the modification of the grazing regime, do not affect to such a great extent to these organisms living in microhabitats. Also, the spatial resolution of soil biological responses is higher than that of aboveground plant responses, implying that a more intensive sampling strategy might be needed to reveal microbial responses. Besides, two years of absence of grazing might be a too short period of time to produce consistent changes on soil microbial community composition. The strong influence of habitat types may also have obscured more subtle changes in microbial composition in our study.

From a meta-analysis, Andriuzzi and Wall^12^ found that the responses of soil biota abundance and activity to herbivores vary by (i) climate, (ii) ecosystem type and (iii) herbivore identity. Our contribution to this list of variables would be (iv) the type of soil organisms studied. According to the results obtained here, we can speculate that the mesofauna are less negatively affected by compaction and the absence of plant litter than the macrofauna. In respect to the microbial communities, it seems that herbivore activity affected more bacterial communities than fungal ones in general, as their richness increased in the absence of grazing. This may be related to the fact that highly grazed grasslands are dominated by bacteria^15^.

### Links between aboveground and belowground prokaryotic and fungal communities (Q2)

In terms of diversity, there was a strong correlation between belowground prokaryotic and fungal communities (consistently with Lanzén *et al*.^23^). However, plant and prokaryotic richness only correlated in grazed plots. This might indicate a stronger dependence of plants on belowground prokaryotes under a stress situation, such as the presence of grazing by large herbivores.

As opposed to alpha-diversity, a strong compositional correlation was found among all communities. Several authors have found a correlation between plant and soil microbial communities, such as Millard and Singh^24^ who suggested that while plant community composition can drive fungal community composition, bacterial community structure is more influenced by SOM quality, hence driven indirectly by plant community composition via plant biomass.

Finally, the strength of these links in terms of alpha-diversity and composition between all studied communities was not found to be affected by grazing.

### Effect of other variables on the measured parameters (Q3)

Rather than grazing, habitat type (and divergence between valley and mountain habitats) better explained differences in community structure. Valley habitat plots were consistently more diverse, especially for prokaryotes. Further, fungal communities (Archaeorhizomycetales, Mortierelalles and Agaricales orders in particular) differed greatly depending on habitat type. This is expected, since aboveground plant and belowground microbial communities are structured according to the edaphoclimatic conditions of a particular site^25^.

Interestingly, the different responses observed to the absence of grazing in the highly acidic mountain sites (increased richness of spore morphotypes) and in the more productive valley sites (reduced Shannon’s diversity from CLPPs) agree with the general predictive framework proposed by Bardgett & Wardle^11^, in which grazing increase soil biological activity in fertile ecosystems, and decrease it in less fertile ones. The significantly increased richness of spore morphotypes found here, and the rather subtle effects detected for other fungal measurements, may also support the hypothesis that predicts stronger aboveground effect on specialist soil organisms^26^ such as AM fungi, that are intimately associated with plant roots.

### Complementarity of methods (Q4)

The determination of CLPPs through Biolog EcoPlates^TM^ appeared more sensitive for detecting community changes caused by grazing abandonment. This may be because it is a method that can detect functional and, therefore, more ecologically relevant aspects of soil microbial communities. This is consistent with a study by Yang *et al*.^27^, using the functional gene array GeoChip 4.0, which indicated that the functional structure of soil microbial communities was very sensitive to the impact of livestock grazing. However, a disadvantage of Biolog^TM^ is the exclusion of organisms that cannot be cultivated, and a bias to fast-growing opportunists that can. However, we recommend the use of Biolog EcoPlates^TM^ to complement the molecular determinations of soil microbial diversity, as they have previously shown repeatability, discriminating power, and sensitivity for many environmental factors^28^.

Determination of diversity of AM spore morphotypes (H’ in particular) showed divergent results compared to those obtained with amplicon sequencing. Few studies have concurrently analysed mycorrhizal diversity in different propagule types^29^. It was not surprising that amplicon data would provide a very different picture of Glomeromycota diversity, since it includes all DNA in a soil sample including which would form spores as well as mycelium, whereas morphological characterisation focuses only on sporulation during a certain season^30^. Morphological characterisation of arbuscular mycorrhiza is a time-consuming, expertise-requiring technique that, based on our results, we cannot recommend to complement molecular methods since it did not add any complementary insights into the communities studied.

## Conclusions

In response to Q1, the cessation of grazing affected plant communities, but also various communities of soil organism in a different way, probably due to the different habitat and trophic level of each community. In the short term, the absence of large herbivores decreased plant diversity and significantly changed its species composition (increasing the *Erica vagans* shrub and the unpalatable *Brachypodium pinnatum*, and decreasing others). Meanwhile, although an increase of macrofauna richness, prokaryotic richness and the abundance of some fungal taxa (Chaetothyriales and Eurotiales), as well as a decrease of the mesofauna QBS index, was detected, the overall belowground microbial community composition did not appear significantly affected. This might be because soil microorganisms operate at much smaller spatial scales and are only indirectly affected by grazing.

In general, the links between plant communities and soil biological communities did not differ in the presence *vs*. the absence of grazing (Q2). Prokaryotic and plant diversity significantly correlated in grazed but not in non-grazed plots, although grazing did not appear to modify the strength of the links between aboveground and belowground communities.

There was an influence of bedrock and habitat type on the observed effects, since more beneficial and negative effects of the absence of grazing were observed in the more fertile and infertile sites, respectively (Q3). Habitat type (especially mountain *vs*. valley) appeared to represent the strongest influence on both above- and belowground communities.

Finally, our results support the use of Biolog EcoPlates^TM^ as a complementary technique to more advanced molecular approach when studying soil microbial communities (Q4).

As a general lesson, and with the final objective of elucidating the consequences of disturbances such as grazing or its absence for ecosystem functioning, the results obtained here bring out the need to consider the complex interactions between aboveground and belowground communities and the effects produced on different soil taxa.

## Methods

### Study area and experimental design

This study was carried out in Gorbeia Natural Park (43°N 2.5°W, 21,016 ha), Basque Country (Spain), in an area traditionally ranged by mixed and unguarded livestock. The dominant large grazers in this area are sheep, cows and horses, with an average stocking rate of 0.75 livestock unit ha^−1^ ^31^. The climate is humid temperate, with an annual mean temperature of 10 °C and a mean precipitation of 2,000 mm. Within the Gorbeia Natural Park, 4 locations with different elevation (mountain sites: 630–720 m altitude; valley sites: 240–410 m altitude), pasture production, vegetation type and parent material were chosen (Table 5). Four permanent exclosures of 10 × 10 m were placed in each site (Table 5).

**Table 5:** Sample overview.

Site	Location	Parent material	Habitat code^*^	Pasture production**	Replicates^***^
Oderiaga	Mountain	Siliceous	6230a (species-rich *Nardus* grasslands, subtype a)	9.2	4
Usotegieta	Mountain	Siliceous	6230c (species-rich *Nardus* grasslands, subtype c)	6.0	4
Arimegorta	Mountain	Calcareous	6170 (Alpine and subalpine calcareous grasslands)	9.5	4
Ipiñaburu-Urigoiti	Valley	Calcareous-Siliceous	-	16.5	4

^*^According to the European Commission Habitats Directive^54^.

^**^Average t ha^−1^ per year.

^***^Number of 10 × 10 m permanent exclosures and corresponding grazed controls established.

### ***In situ*** vegetation and soil analyses

Two years after the establishment of the exclosures, plant richness and percentage cover were determined by randomly locating a 50 × 50 cm square (5 times per sampling area; an average of these measurements was used hereinafter). Root abundance and root depth were measured *in situ* following Mijangos *et al*.^32^. Compaction was measured using a digital penetrometer (Rimik CP40II) to record the pressure needed to penetrate the soil over a 0–75 cm depth profile. Water infiltration capacity was determined by quantifying the time needed to absorb 230 ml of water poured into a 10 cm diameter tube fixed to previously water-saturated soil. Soil CO_2_ emissions were determined with an infrared gas analyser (EGM-4, PP Systems, Amesbury, MA, USA) linked to a cylindrical soil respiration chamber (SRC-1, PP-Systems) as described in Biau *et al*.^33^. Finally, morphotypes of macrofauna (e.g., worms, cockroaches, woodlice, millipedes, centipedes, earwigs, ants, termites, grasshoppers, beetles, true bugs, spiders, snails, cicadas) were also counted within a block of soil 30 cm deep × 25 cm long × 25 cm wide^23,32^. All these measurements were performed inside and outside (in an area of similar size immediately next to the exclosures) the 16 exclosure areas (32 plots in total).

### Soil sampling and analyses

On the same day that *in situ* measurements were taken, a composite soil sample of topsoil (0–10 cm) was collected using a core soil sampler (25 mm diameter; 15–20 cores taken at random throughout the whole sampling area) and sieved to < 2 mm (except for the determination of mesofauna). Subsequently, soil humidity was determined. For the measurements of physicochemical parameters, soil samples were air-dried at ambient temperature until constant weight. Soil organic matter content, pH, total nitrogen, Olsen phosphorus and extractable potassium were measured according to standard methods^34^.

For biological parameters, soils were stored fresh at 4 °C for a maximum of one month until analysis. Basal and substrate-induced respiration (SIR) were measured following ISO 16072 Norm^35^ and ISO 17155 Norm^36^, respectively. The mesofauna QBS index, where microarthropods are separated according to convergent morphological features to evaluate the level of adaptation to the soil environment, was calculated following Parisi *et al*.^37^. CLPPs of cultivable heterotrophic bacteria were determined with Biolog Ecoplates^TM,^ ^38^, where measurements corresponding to an incubation time of 48 h were chosen for further calculations, following Epelde *et al*.^39^. Arbuscular mycorrhizal (AM) spores were isolated from 50 g dry weigh (DW) of soil from each sample by wet sieving and decanting. After manually selecting all the AM spores present under a microscope (40x), they were mounted on slides in either polyvinyl alcohol – lactic acid – glycerol (PVLG) or a mixture of PVLG – Melzer reagent. Spore characteristics were observed under a microscope at 200x and 400x for the establishment and quantification of morphotypes.

Soil sub-samples for molecular analysis were stored at −20 °C. DNA extraction and amplicon library preparation were carried out as described in Lanzén *et al*.^23^. Briefly, PowerSoil DNA Isolation kits were used for extraction (Mo-Bio Laboratories, Carlsbad, CA, USA); primers 515F (CTGNCAGCMGCCGCGGTAA) and 806R (GGACTACHVGGGTWTCTAAT) used for amplification of prokaryotic communities, and ITS1F (CTTGGTCATTTAGAGGAAGTAA^40^) and ITS2R (GCTGCGTTCTTCATCGATGC^41^) for fungi, using a dual-indexed adapter-linked protocol based on Caporaso *et al*.^42^. Library preparation and Illumina MiSeq V2 sequencing was carried out at StarSEQ GmbH, Mainz, Germany. Sequence data has been deposited to the European Nucleotide Archive with study accession PRJEB9654, under sample group “Biopasto”.

### Sequence data analysis

Sequence data processing and taxonomic classification was performed as described in Lanzén *et al*.^43^. Briefly, overlapped read-pairs from 16 S rRNA amplicons were quality-filtered and overlapped using *usearch* ^44^ and truncated to a length of 253 nt, and ITS read-pairs trimmed to remove the reverse primer using *cutadapt* ^45^. All quality-filtered overlapped sequences from 16S rRNA and ITS amplicons were clustered into OTUs using a 3% divergence cut-off using *vsearch* ^46^.

Representative prokaryotic and fungal OTU sequences were aligned to the SilvaMod v106 and UNITE reference databases, respectively, using *blastn* (v.2.2.25 + task megablast), and taxonomically classified using CREST with default parameters^47^. The relative taxon abundances derived by CREST at order rank were used in further analysis. This approach was motivated by the fact that sequence-based relative abundance has been demonstrated to provide meaningful semi-quantitative information when comparing community structure between samples^48^, in spite of being affected by issues such as ribosomal copy number variability and preferential amplification. Taxa of lower ranks detected that lacked child nodes at order level were manually included into the respective dataset.

### Statistical analysis

In every community composition dataset (plants, CLPPs of bacteria, AM spore morphotypes, amplicon sequences of 16S rRNA and ITS), two measures of diversity were calculated: species richness (S) and Shannon’s diversity index (H’)^49^. In the case of CLPPs of bacteria, the number of utilized substrates (*i.e*., the number of substrates with an absorbance value > 0.25) was used equivalent to S; H’ was calculated considering absorbance values at each well as equivalent to species abundance. For amplicon sequence data, rarefied richness estimates, interpolating the expected richness at the lowest sample-specific sequencing depth, were used to compensate for variation in read depth across samples, using the R package *vegan* ^50^. Pearson correlation analyses were carried out in order to compare these diversity indexes.

To compare grazed *vs*. non-grazed plots on all measured plant and soil parameters, linear mixed-effect models were used as implemented in the R package *nlme* (*lme* function^51^), including grazing as fixed effect and site as random effect. In order to study the influence of grazing at the community composition level, partial canonical correspondence analyses (pCCA) were performed with compositions as response variables, and including grazing as an explanatory variable and site as a covariable. Likewise, in order to study the influence of other explanatory variables besides grazing, such as habitat and bedrock, redundancy analyses (RDA) were performed following forward selection of the subset of explanatory variables that significantly explained the variation in community composition and other response variables. The statistical significance of all canonical axes was also tested by means of non-parametric Monte Carlo permutation test. All multivariate analyses were done using Canoco 5^52^. Mantel-tests were performed to compare community composition between Bray-Curtis similarity matrices, using the program PAST^53^.

## Electronic supplementary material

Supplementary information
