# Supplementary material for: Short-term effects of non-grazing on plants, soil biota and aboveground-belowground links in Atlantic mountain grasslands
Source: Sci Rep. 2017 Nov 8;7:15097. doi: 10.1038/s41598-017-15345-1 (PMC5678074; doi:10.1038/s41598-017-15345-1)
Supplement: Supplementary file 1 — Supplementary information [file 41598_2017_15345_MOESM1_ESM.pdf]

Short-term effects of non-grazing on plants, soil biota and aboveground-belowground links in Atlantic mountain grasslands

Lur Epelde[1*], Anders Lanzén[1], Iker Mijangos[1], Estibaliz Sarrionandia[2], Mikel Anza[1], Carlos Garbisu[1]

Supplementary Table S1. Summary of the linear mixed-effect models on the 20 most abundant taxa in the plant, prokaryotic and fungal communities. Statistically significant results (p-value < 0.05) appear in bold.

Plants			Prokaryotes			Fungi		
Species	t-value	p-value	Order	t-value	p-value	Order	t-value	p-value
Agrostis capillaris	-0.095	0.925	Acidobacteriales	-1.838	0.078	Archaeorhizomycetales	-0.710	0.484
Festuca gr. rubra	0.282	0.781	Planctomycetales	0.297	0.769	Mortierellales	0.474	0.639
Agrostis curtisii	**-3.145**	**0.004**	Chthoniobacterales	0.273	0.787	Agaricales	0.010	0.992
Galium saxatile	-0.614	0.545	Rhizobiales	0.533	0.598	Pezizales	0.170	0.867
Holcus lannatus	1.749	0.092	Ktedonobacterales	-0.224	0.825	Filobasidiales	-0.812	0.424
Potentilla erecta	-0.432	0.670	Frankiales	-0.185	0.855	Hypocreales	0.791	0.436
Brachypodium pinnatum	**2.598**	**0.015**	Xanthomonadales	-0.976	0.338	Helotiales	-0.336	0.740
Trifolium repens	**-3.165**	**0.004**	Sphingobacteriales	-0.226	0.823	Sordariales	-0.746	0.462
Danthonia decumbens	**-3.290**	**0.003**	Rhodospirillales	1.736	0.094	Glomerales	-0.260	0.797
Ranunculus repens	2.039	0.052	WD2101 soil group	-0.016	0.987	Saccharomycetales	-0.352	0.728
Lotus corniculatus	1.769	0.089	Myxococcales	0.454	0.653	Pleosporales	-1.504	0.145
Potentilla montana	-1.165	0.255	Bacillales	0.401	0.691	Trichosporonales	1.098	0.282
Carex caryophillea	**-3.582**	**0.001**	WD272	1.723	0.097	Dothideales	-0.008	0.994
Erica vagans	**2.057**	**0.050**	GR-WP33-30	1.002	0.326	Paraglomerales	0.286	0.777
Rumex acetosella	-1.107	0.278	Solirubrobacterales	0.527	0.602	**Chaetothyriales**	**2.110**	**0.045**

Glechoma hederacea L.	-0.832	0.413	Burkholderiales	0.371	0.714	**Eurotiales**	**2.292**	**0.030**
Carex sp.	-1.227	0.231	AKIW543	0.654	0.519	Cantharellales	-0.646	0.524
Plantago lanceolata	-1.118	0.274	Corynebacteriales	-0.126	0.901	Xylariales	-1.369	0.183
Veronica persica	1.305	0.203	Cytophagales	1.029	0.313	Trechisporales	-0.925	0.364
Ranunculus acris L.	1.656	0.110	Nitrosomonadales	-0.329	0.745	Geoglossales	0.570	0.573

Supplementary Table S2. Mantel´s correlations (R statistic) and corresponding p-values, comparing community composition across datasets.

			Plants	Prokaryotes	Fungi	Glomeromycota	CLPPs bacteria	AM spore morphotypes	
All plots		Mantel´s correlation							p-value
	Plants			0.000	0.000	0.000	0.037	0.000	
	Prokaryotes		0.70		0.000	0.000	0.000	0.000	
	Fungi		0.58	0.62		0.000	0.001	0.003	
	Glomeromycota		0.52	0.63	0.39		0.137	0.005	
	CLPPs bacteria		0.15	0.31	0.22	0.13		0.139	
	AM spore morphotypes		0.34	0.37	0.17	0.26	0.09		
Only grazed		Mantel´s correlation							p-value
	Plants			0.000	0.000	0.001	0.002	0.001	
	Prokaryotes		0.78		0.000	0.001	0.002	0.001	
	Fungi		0.81	0.53		0.005	0.022	0.009	
	Glomeromycota		0.42	0.56	0.23		0.336	0.035	
	CLPPs bacteria		0.35	0.39	0.21	0.07		0.168	
	AM spore morphotypes		0.44	0.49	0.26	0.27	0.11		
Only non-grazed		Mantel´s correlation							p-value
	Plants			0.000	0.000	0.000	0.061	0.010	
	Prokaryotes		0.83		0.000	0.000	0.008	0.020	
	Fungi		0.51	0.66		0.004	0.011	0.538	
	Glomeromycota		0.64	0.72	0.27		0.057	0.038	
	CLPPs bacteria		0.21	0.35	0.24	0.28		0.168	
	AM spore morphotypes		0.29	0.25	-0.01	0.28	0.14		

Supplementary Table S3. UTM coordinates of the 16 sampling sites.

Site	Replicate	X coordinate	Y coordinate
Oderiaga	1	511426	4768058
Oderiaga	2	510893	4766822
Oderiaga	3	511421	4766920
Oderiaga	4	511579	4766921
Usotegieta	1	514633	4765144
Usotegieta	2	514589	4765222
Usotegieta	3	514289	4765311
Usotegieta	4	514632	4765363
Arimegorta	1	519423	4766255
Arimegorta	2	519810	4766101
Arimegorta	3	519973	4765910
Arimegorta	4	519976	4765821
Ipiñaburu-Urigoiti	1	520671	4768648
Ipiñaburu-Urigoiti	2	520778	4768493
Ipiñaburu-Urigoiti	3	512463	4770543
Ipiñaburu-Urigoiti	4	512405	4770469

Supplementary Figure Legends

Supplementary Figure S1. Barplots representing the distribution of the 20 most abundant taxa. A: plant community, species level, percentage cover; B: prokaryotic community, order level, relative abundance; C: fungal community, order level, relative abundance. Average values (n = 4).

Supplementary Figure S2. Biplot of the partial canonical analysis performed with plant community composition as response variables, grazing as explanatory variable, and site as covariable (pseudo-F = 2.4, p-value = 0.002). Only the 15 response variables with best fit are shown. Variation explained by the first axis is indicated between brackets.

Supplementary Figure S3. Biplot of the redundancy analysis performed with (A) Glomeromycota community composition at the species level (pseudo-F = 5.7, p-value = 0.002), (B) CLPPs of bacteria (pseudo-F = 2.3, p-value = 0.002), and (C) arbuscular mycorrhizal spore morphotype abundances (pseudo-F = 2.6, p-value = 0.002) as response variables, with grazing, bedrock and habitat as explanatory variables. Variation explained by each axis is indicated between brackets. The explanatory variables that appear are those that significantly explained the variation in response data following forward selection; the contribution percentage of each variable is shown between brackets.

A)

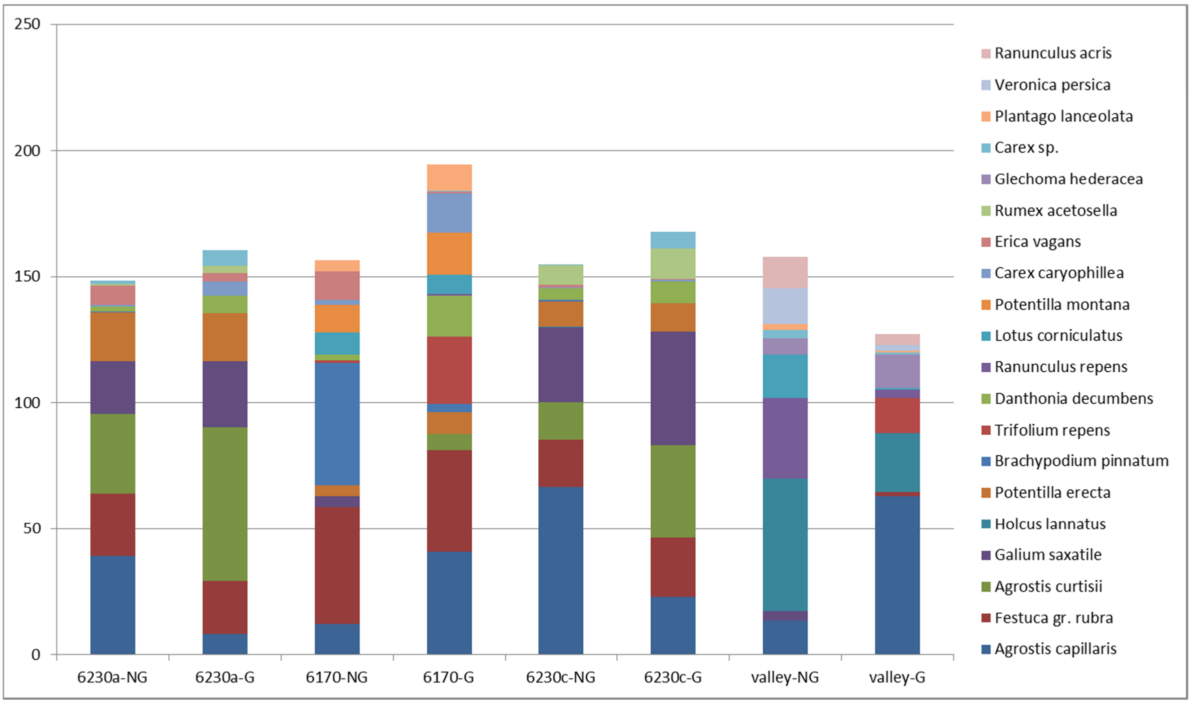

250
200
150
100
50
0
6230a-NG
6230a-G
6170-NG
6170-G
6230c-NG
6230c-G
valley-NG
valley-G
Ranunculus acris
Veronica persica
Plantago lanceolata
Carex sp.
Glechoma hederacea
Rumex acetosella
Erica vagans
Carex caryophillea
Potentilla montana
Lotus corniculatus
Ranunculus repens
Danthonia decumbens
Trifolium repens
Brachypodium pinnatum
Potentilla erecta
Holcus lannatus
Galium saxatile
Agrostis curtisii
Festuca gr. rubra
Agrostis capillaris

B)

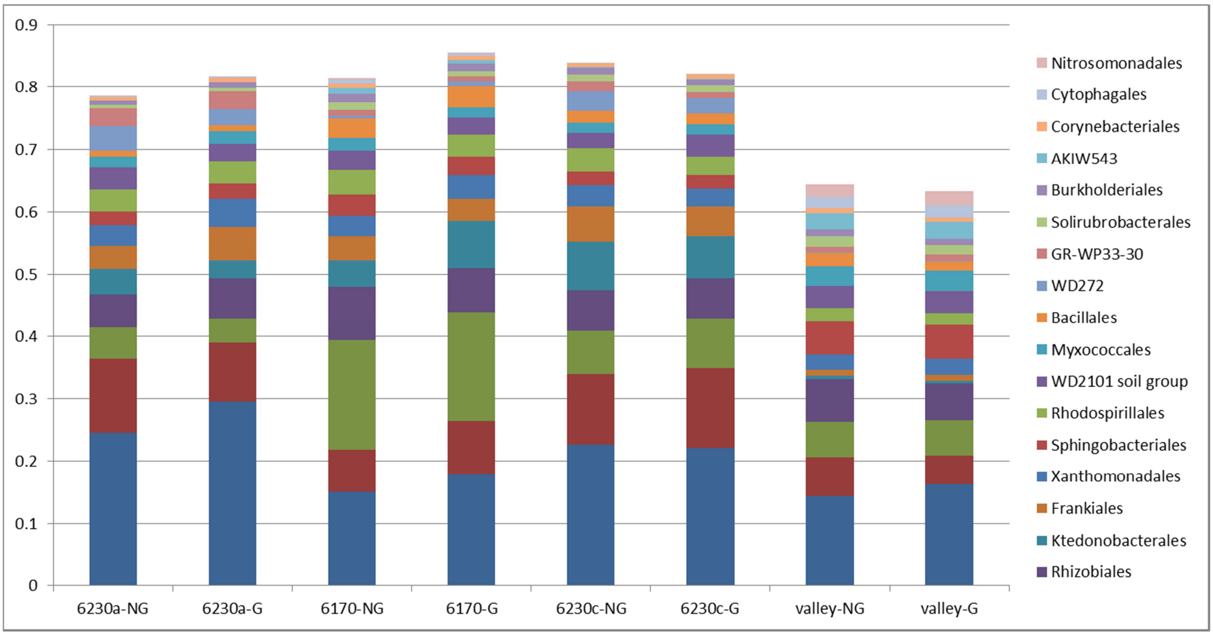

0.9
0.8
0.7
0.6
0.5
0.4
0.3
0.2
0.1
0
6230a-NG
6230a-G
6170-NG
6170-G
6230c-NG
6230c-G
valley-NG
valley-G
Nitrosomonadales
Cytophagales
Corynebacteriales
AKIW543
Burkholderiales
Solirubrobacterales
GR-WP33-30
WD272
Bacillales
Myxococcales
WD2101 soil group
Rhodospirillales
Sphingobacteriales
Xanthomonadales
Frankiales
Ktedonobacterales
Rhizobiales

C)

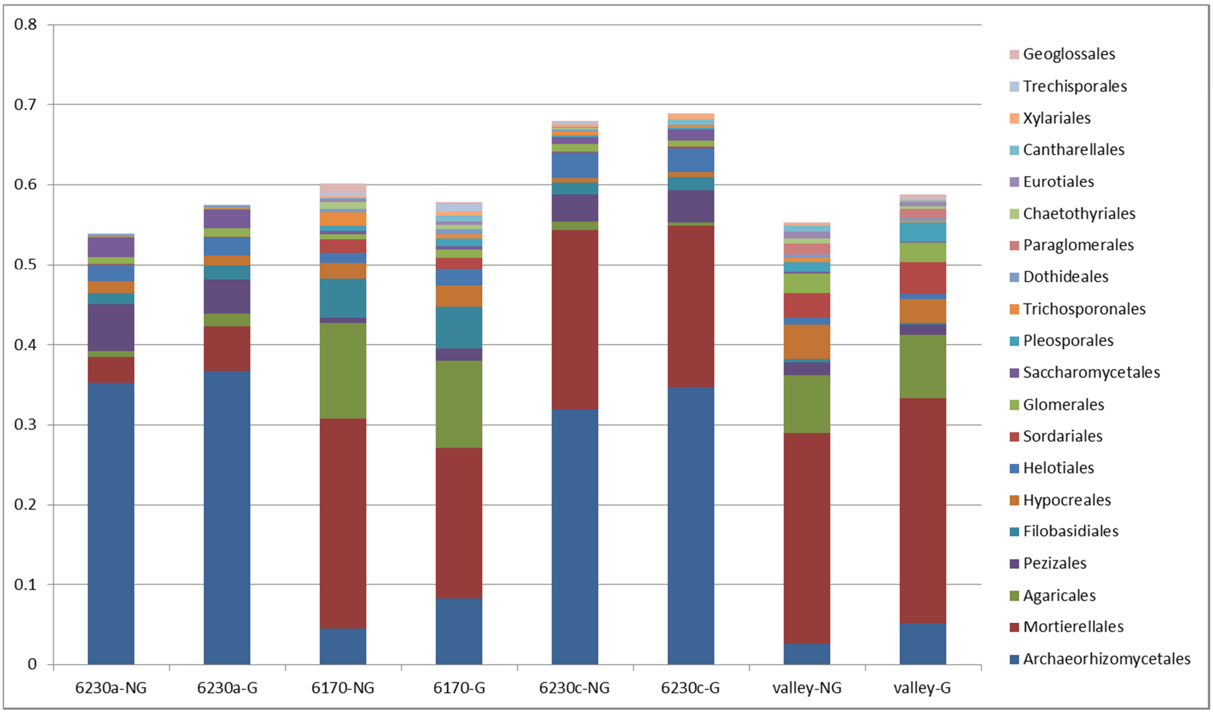

SUPPLEMENTARY FIGURE S1

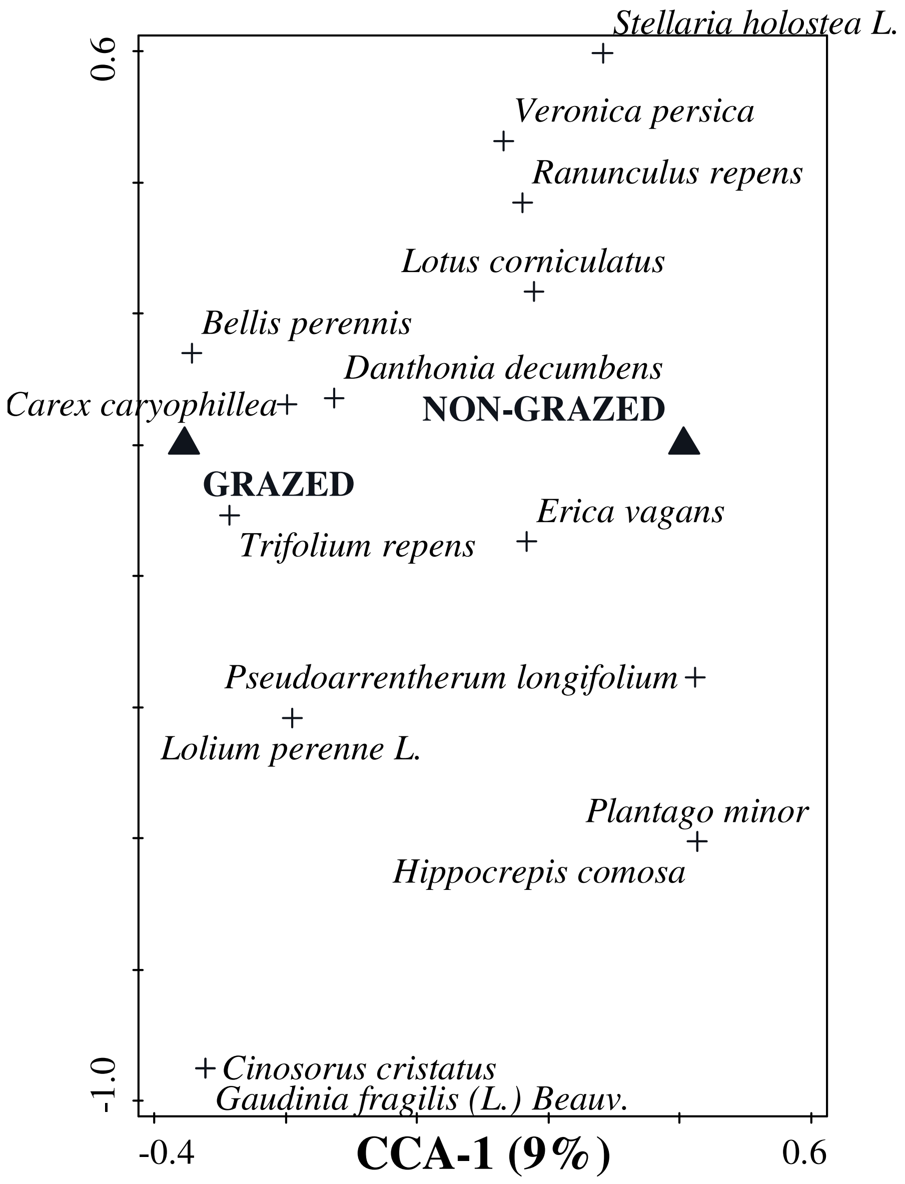

SUPPLEMENTARY FIGURE S2

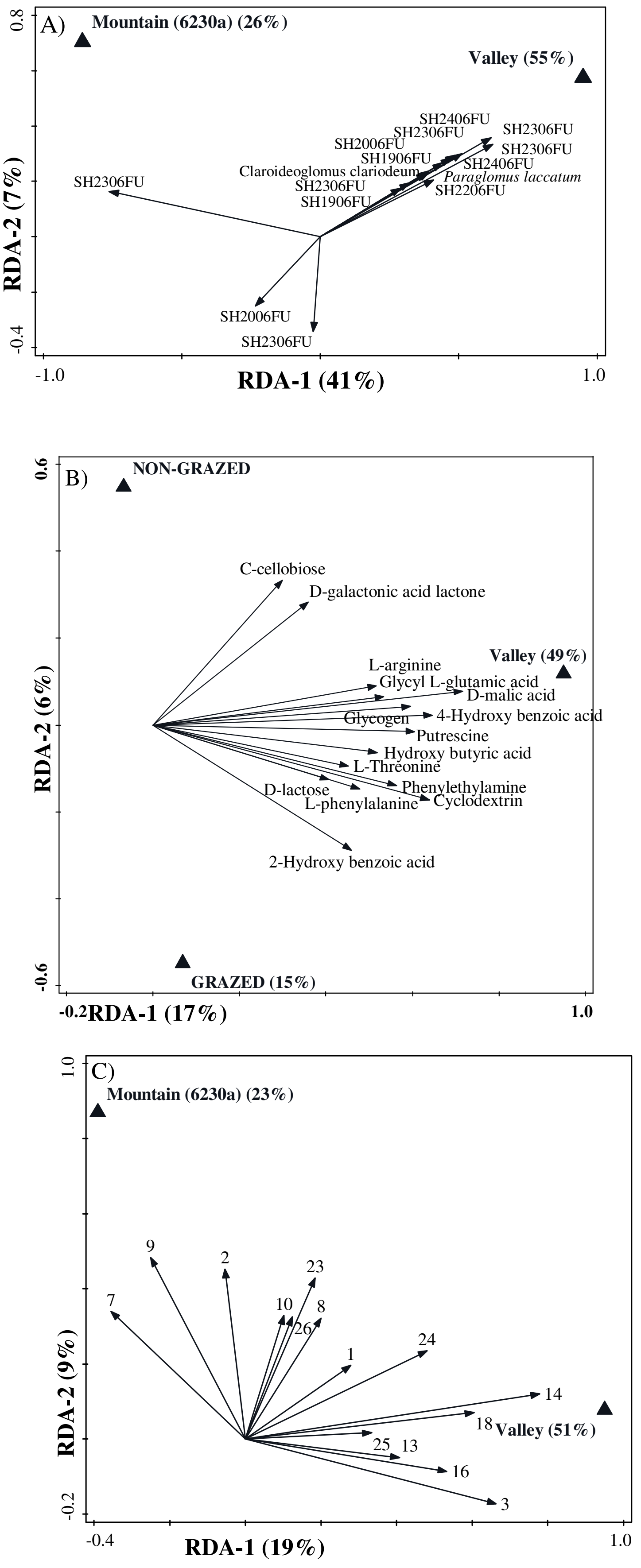

SUPPLEMENTARY FIGURE S3
